# Electrical and Thermal Properties of Heater-Sensor Microsystems Patterned in TCO Films for Wide-Range Temperature Applications from 15 K to 350 K

**DOI:** 10.3390/s18061831

**Published:** 2018-06-05

**Authors:** Ryszard Pawlak, Marcin Lebioda

**Affiliations:** Institute of Electrical Engineering Systems, Lodz University of Technology, Lodz 90-924, Poland; marcin.lebioda@p.lodz.pl

**Keywords:** transparent conducting oxides, laser patterning, heating microstructures, cryogenic

## Abstract

This paper presents an analysis of the electrical and thermal properties of miniature transparent heaters for use in a wide range of temperature applications, from 15 K to 350 K. The heater structures were produced in transparent conducting oxide (TCO) layers: indium tin oxide (ITO) and ITO/Ag/ITO on polymer substrates-polyethylene naphthalate (PEN) and polyethylene terephthalate (PET), by direct laser patterning. Thermo-resistors for temperature measurement were created in the same process, with geometry corresponding to the shape of the heating path. The thermo-resistors integrated with the heating structure allowed easy control of the thermal state of the heaters. Laser patterning provided high precision and repeatability in terms of the geometry and electrical properties of the heater-sensor structures. Measurements at temperatures from 15 K to above room temperature (350 K) confirmed the excellent dynamics of the heating and cooling processes, due to current flow. The largest value for surface heating power was over 3 W/cm^2^. A heater-sensor structure equipped with a small capacity chamber was successfully applied for controlled heating of small volumes of different liquids. Such structures have potential for use in research and measurements, where for various reasons controlled and accurate heating of small volumes of liquids is required.

## 1. Introduction

One of the special applications of transparent conductors is as invisible joule heaters. The development of transparent heaters was summarized recently in [1]. Transparent heaters can be made from metal oxides, oxide nanoparticles [2], carbon nanotubes layers [3,4,5], graphene [6,7,8], metal nanowire networks [9,10,11], metal meshes or various composites of these materials [12,13,14,15]. Heaters based on transparent conductors are used in liquid-crystal display (LCD), disposable medical devices, and in many areas of research. Transparent heaters are developing advantages over hydrophobic coatings for defrosting and defogging.

Oxide conductors (in this case, indium tin oxide or ITO) were probably used for the first time as surface heaters in a temperature-controlled perfusion apparatus for electrophysiological studies [16]. An ITO layer was deposited on a glass sheet to preheat the perfusion solution. Micro-sized ITO heaters deposited on glass were applied for temperature control in a system for transcription and translation of DNA gene sequences [17]. Surface heaters made of ITO have been used widely in research on boiling phenomena. An ITO layer deposited on glass was used to ensure uniform surface heating in studies of bubble dynamics in R-123 coolant [18]. A flat ITO heater was used as a transparent thermal source in a microchannel chip for research into polymerase chain reactions [19]. In [20], the authors reported the results of research into heat transfer in glass microtubes with different shaped cross-sections. The outer surfaces of the tubes were covered with an ITO/Ag heating layer. Studies on boiling phenomena during the flow of liquid nitrogen in a mini-tube covered by an ITO heater were reported in [21]. A test stand for flow pattern characterization of R134a refrigerant in a minichannel used a similar configuration [22]. An ITO microheater on glass was used to build a microchip for research into polymerase chain reactions and quantitative analysis of DNA amplicons [23]. For visualization of boiling phenomena in a pool of saturated water [24], an ITO heater was deposited on a sapphire substrate. An ITO layer acting as the resistive heating element and simultaneously as the electrical ground on a sapphire tube allowed visualization of the flow patterns in experiments on heat transfer augmentation, when an electric field was applied in two-phase heat exchangers [25]. Another study, which focused on the nucleation and dynamic behavior of bubbles in refrigerant R113 [26], created the bubbles using an ITO plate heater in the presence of a nonuniform electric field. A cost competitive solution compared to depositing ITO layers by sputtering was proposed in [2]. A layer of heaters was formed by spin-coating an ITO nanoparticle solution on a glass substrate, followed by thermal sintering. Experimental studies on the enhancement of heat transfer on the patterned surface of an ITO heater were reported in [27].

A further application for transparent oxide heaters is as sensors. Programming or modulating temperature is one way to obtain better selectivity and sensitivity in semiconductor gas sensors. A miniature ITO resistor was developed as a platform for thin film gas sensors [28]. A zinc oxide doped with Ga (GZO) film was investigated as a candidate for replacing ITO film as a transparent heater in [29]. Recently, technology for the continuous sputtering of ITO/Ag/ITO film was demonstrated [30]. The heating properties of this multilayer were confirmed. Transparent oxide heaters have found interesting applications in some microsystems. Thermopneumatic-actuated microvalves with a layer of ITO microheaters deposited on a glass surface were presented in [31]. A very useful micropump for lab-on-a-chip systems, driven thermally by an ITO microheater, was demonstrated in [32]. An integrated microfluidic system for micro-total analysis systems (micro-TAS), containing a Paraffin-actuated microvalve and a micropump with an ITO heater, was successfully developed in [33,34]. Most commercially available and upcoming systems for defogging and deicing applications in aircraft and automobiles demand transparent ITO layer heaters covering large areas [35,36].

The examples of the use of transparent oxide as heaters discussed so far and summarized in Table 1 justify the following observations:

- the heaters were deposited mainly on glass [2,16,17,18,19,20,21,22,23,26,27,29,31,32,33,34] and other solid dielectric materials, such as sapphire [25] or silicon nitride [28]. Only ITO/Ag/ITO layers were deposited on a polymeric substrate [30];
- the heaters mostly covered large surface areas and were embedded directly on the surface of the heated object [2,19,20,21,22,24,25,26]. The heaters, with dimensions of less than a few millimeters, were made using complex technologies (masking, photolithography, or etching) [17,23,27,28,29,31,32,33,34];
- almost all of the heaters had simple geometric shapes: square, rectangle, and a few were formed to meander [15,33,34].

In this work, we propose an analysis of transparent heaters formed in an ITO or ITO/Ag/ITO layer integrated with temperature sensors. Both the transparent heaters and the temperature sensors are produced by laser ablation in a single technological process. The ITO and ITO/Ag/ITO layers were on a polymer substrate. The properties of these materials, including at cryogenic temperatures, were investigated in previous works [37,38]. Both the heaters and the sensors can be given various kinds of shapes and transverse dimensions, down to about 40 µm. The structures can be multiplied, preserving the assumed parameters. Laser shaping structures in oxide layers of nanometer thickness was demonstrated in [38,39]. The proposed solution could be useful for controlling the temperature of small-scale cryogenic systems, especially to rapidly form a temperature gradient over a short distance. It is possible to multiplex the microstructure, creating a dedicated matrix to precisely control local temperature. One of the main features of the proposed system is its very low thermal capacity. Our technology also enables rapid planning and implementation. Designing the heater-sensor structure, producing the microstructures with electrodes, and lamination takes less than 2 h. The proposed method for producing microheaters could be useful for rapid prototyping in various research areas, including chemistry, biology and biomedicine.

## 2. Experiments

### 2.1. Materials and Structures

**Figure 1:**
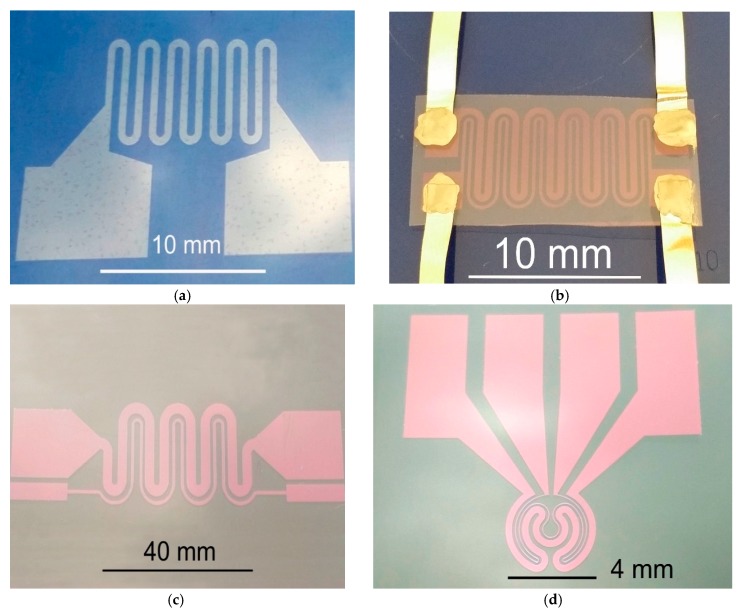
Samples of structures made by direct laser patterning in indium tin oxide (ITO) and ITO/Ag/ITO layers: (**a**) meander shaped resistor (ITO layer on PET); (**b**) resistor with thermosensor (ITO/Ag/ITO layer on PET); (**c**) resistor with thermosensor—“long” (ITO/Ag/ITO layer on PET); (**d**) microheater with sensor (ITO/Ag/ITO layer on PET).

Two different transparent layers were used. An ITO layer with a thickness of 125 nm and surface resistance 15 Ω/sq was deposited on a polyethylene naphthalate (PEN) substrate (150 µm). ITO/Ag/ITO multilayers with respective thicknesses of 70/10/70 nm and surface resistivity 4 Ω/sq were deposited on a polyethylene terephthalate (PET) substrate with a thickness of 140 µm. The efficiency and precision of the method of producing structures with different geometries and dimensions in transparent conductive layers by direct laser writing using nanosecond laser pulses had been confirmed in previous studies [37,38,39]. The heaters and sensors in the current research were formed using a single-mode fiber laser redENERGY G3 SM 20 W (SPI Lasers, Ltd., Southampton, UK), which provides a high-quality beam (M^2^ < 1.3). The laser beam was scanned using a 2-Axis Scan Head (Xtreme, Nutfield Technology, Inc., Hudson, NH, USA) equipped with an F-theta lens of 100 mm and controlled by SB-1P Waverunner software. The optimal process parameters, namely pulse energy, pulse duration, pulse repetition frequency, and scanning velocity were determined experimentally, as 150 µJ, 25 ns, 80 kHz, 800 mm/s for the ITO layer and 120 µJ, 25 ns, 72 kHz, 800 mm/s for the ITO/Ag/ITO layer. A schematic diagram for producing the integrated structures of the heater-sensors is given in [39]. Samples of structures made in ITO and ITO/Ag/ITO layers by laser micromachining are shown in Figure 1.

**Figure 2:**
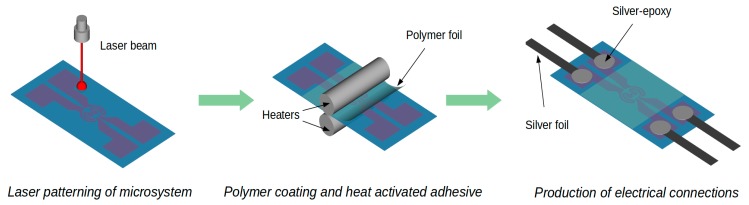
Preparation of heater-sensor structures.

The electrical connections to the contact pads of the heater and sensor (Figure 1b–d) were made using thin Ag foil (35 µm) and electrically conductive silver-epoxy Elpox AX 15s [40]. The integrated heater-sensor structures were protected by lamination with thin (60 µm) PET foil (Figure 2). To examine their functional properties, such as the ability to heat a defined volume of liquid and the distribution of heat close to heater surface, some of the structures were made with microchambers laminated to the surface.

### 2.2. Experimental Procedures

**Figure 3:**
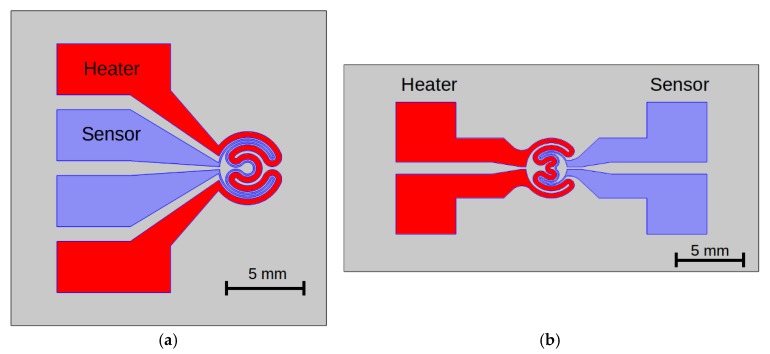
Geometry of heater-sensor structures: (**a**) structure with one-sided leads; (**b**) structure with two-sided leads.

The main research work was focused on measurement and analysis of the dynamics of electrical and thermal processes in thin-film microsystems fabricated using ITO and ITO/Ag/ITO layers. Structures with two different geometries were used (Figure 3). The shape of the heating area was determined by expected application of presented structures, e.g., heating of liquid drops in biological and biomedical experiments. A circular heating area provides uniform heating, so heating paths were combined from sections of coaxial rings. Generally, the experimental study was conducted in two modes of heat transfer, defined by environmental conditions:

- natural convection: the investigated structure was suspended under natural convection conditions at room temperature. Convection and radiation were observed. Microsystems with microchambers attached to the surface were also investigated. The microchambers were filled with liquid as an additional heat load for the heating structure;
- heat conduction at cryogenic temperatures (295–15 K). The structure was mounted on a copper heat exchanger in a cryogenic system. The investigation was conducted in a vacuum chamber equipped with a radiation shield.

The heating process was first studied at room temperature under natural convection conditions. This enabled analysis of the most typical and natural configurations for heat transfer. The thermal capacity and conductivity of the structure played key roles in this configuration, enabling analysis of dynamic of thermal processes. Under natural convection conditions, the structure was suspended using silver electrodes. The main part of the microsystem did not have thermal contact with solid state objects. These assumptions allowed the effect of heat convection and radiation to be observed.

**Figure 4:**
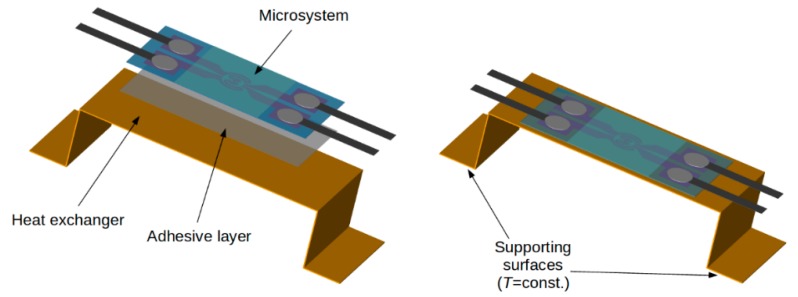
Integration of structure and heat exchanger for cryogenic research.

To study the second mode of heat transfer, the structure was mounted on a heat exchanger and thermally isolated for cryogenic studies using a vacuum chamber and radiation shield [37]. Heat transfer to the heat exchanger was monitored. These studies at cryogenic temperatures established the range of temperatures within which the structures remained usable. Thin copper foil (100 µm) was used to format the copper heat exchanger. The thin copper foil provided high thermal conductivity and relatively small thermal capacity as a result of its low mass (~3.5 g). The two ends of the exchanger were mounted on the cold finger (second stage) of a cryocooler. This provided accurate temperature control (Figure 4). A thin polymer adhesive layer was used to mount the structure on the exchanger. It should be noted that the copper heat exchanger was an additional heat load for the heating structure and its small cross-section and significant length allowed observation of the longitudinal temperature gradient. In addition, this configuration enabled observation of how an additional heat load changed the dynamics of the thermal processes while also testing a potential application of the heating structures.

**Figure 5:**
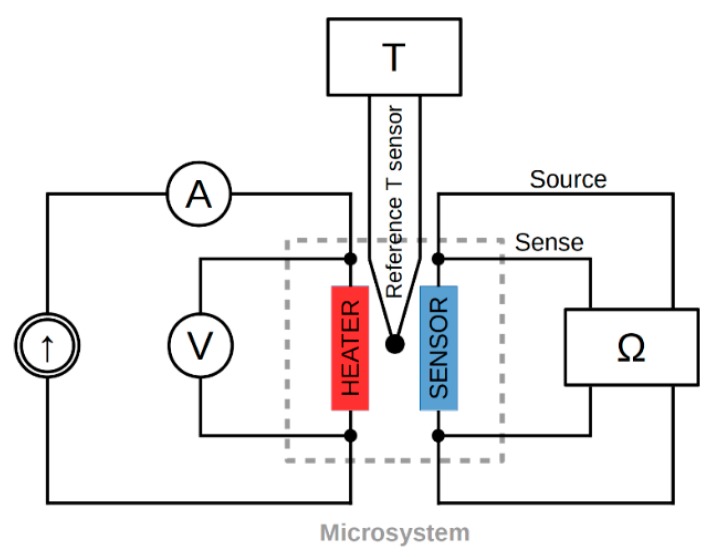
Diagram of the measurement system.

The same experimental procedures and instrumentation were used in both studies (Figure 5). Power to the heater was supplied by a controllable current source. Current and drop voltage were measured by two digital HP34401A multimeters (Keysight Technologies, Santa Rosa, CA, USA). The results enabled estimation of the power of joule heating. The four probe method and a digital HP34420A ohmmeter (Keysight Technologies, Santa Rosa, CA, USA) were used to measure the resistivity changes in the integrated temperature sensor. The temperature of the structure in the cryogenic system was measured using a Lake Shore 331 (Lakeshore Cryotronics Inc., Westerville, OH, USA) temperature controller and a silicon diode DT-670-SD (Lakeshore Cryotronics Inc., Westerville, OH, USA) reference sensor. The controller was part of the cryogenic system. It controlled and stabilized the temperature of the second stage of the cryocooler. The samples examined at room temperature were equipped with a miniature thermocouple to measure the temperature of the central point in the structures. A copper-constantan thermocouple was permanently integrated with the structure by the polymer coating process. Using a thin wire thermocouple (140 µm) avoided the need for a heat sink in the structure. A computer measurement system was used to control the measurement procedure and data acquisition process.

## 3. Results and Discussion

### 3.1. Room Temperature

**Figure 6:**
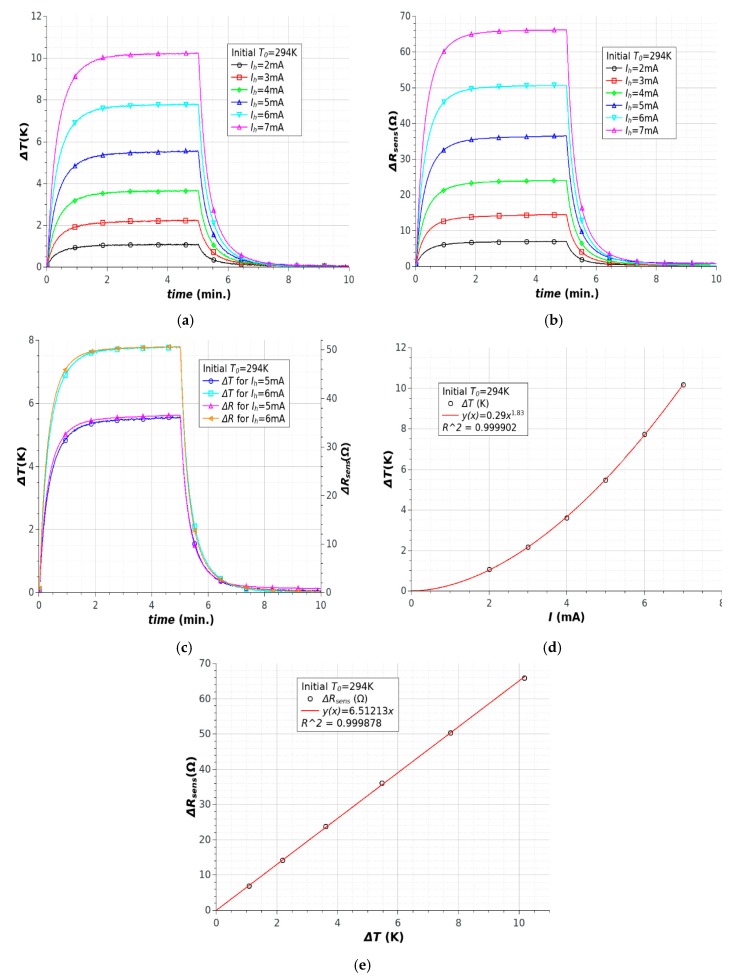
Heating of microstructures at room temperature: (**a**) temperature changes; (**b**) sensor resistance changes; (**c**) relationship between temperature changes and sensor resistance changes during heating process; (**d**) temperature changes due to heater current value; (**e**) relationship between sensor resistance changes and temperature changes.

The time dependences for temperature changes ΔT and sensor resistance changes ΔR are shown in Figure 6a,b. All results confirmed that the processes for heating and cooling were typical for natural convection conditions. Increasing the current caused the temperature to rise. The changes were proportional to the current squared (Figure 6d), which is a typical relationship for the joule heating process. This was confirmed by the high temperature increase for higher currents (ΔT = 2 K for I_h_ = 3 mA, ΔT = 8 K for I_h_ = 6 mA). The elongation of temperature stabilization time for higher currents can result from changes in the resistance of the heater and especially from the character of the heat transfer processes. Changes in heater resistance were observed in the time dependence of heat power, especially for higher currents (Figure 6b). The main advantage of the microstructures is the short time constant for heating and cooling processes. It should be noted that the character of the heating and cooling processes was the same for all currents, which is important from the perspective of potential applications.

The actual time constants were probably smaller than those estimated on the basis of experimental results due to the thermal capacity and conductivity of the reference temperature sensor (a copper-constantan thermocouple). Strong thermal coupling between the heater and the sensor mounted in the structure was observed (Figure 6c). This advantageous feature resulted in the rapid reaction of the integrated sensor to temperature changes, which was similar to the results obtained using thermocouple measurements. It should be noted that the temperature resistivity changes in the heater were the same as those of the sensor. This feature resulted in changes in surface power and a relationship between heater current and surface power which was more important at high temperatures. The heater and sensor were produced from the same material and strong thermal coupling between them was observed. The resistivity changes in the heater were inessential, because direct temperature measurement provided high accuracy and full control of the heating process. 

Sensitivity of sensor depends on its thermal coefficient of resistance. Experimental results showed linear dependence of resistance changes versus temperature changes (Figure 6e). The value of thermal coefficient of resistance, determined on the basis of this relationship, was about 6.9 × 10^−4^ 1/K.

The effective area of a heater with the geometry presented on Figure 3 covered about 50% of the total area of the microstructure and the sensor area about 14.5%. Sensor and heater paths of the same shape ensured high thermal coupling. The thermal power generated by a current of 15 mA in a microsystem with a diameter of 8 mm was about 67.5 mW. In this case, the total surface power was about 0.27 W/cm^2^. It should be noted that, in the presented geometry, the real surface power in the heater path was two times higher. Our structures could withstand a large heat load. No deformations or delamination of the transparent conducting oxide (TCO) layer were observed for higher current values (>100 mA) when the surface power was over 3 W/cm^2^. This was an effect of the high precision of laser micromachining [37,39], which provided high patterning quality without narrowing what could generate hotspots. This technology is useful for creating submillimeter (up to 100 μm) surface structures in thin films (e.g., TCOs) with good quality, also on polymer substrates. Processes based on photolithography allow better quality of structures even in nanoscale, but laser patterning has advantages against photolithography. The process was fast, furthermore the shape and dimensions of structures could be easy modified. No other method provides the possibility for rapid design and prototyping such surface structures.

**Figure 7:**
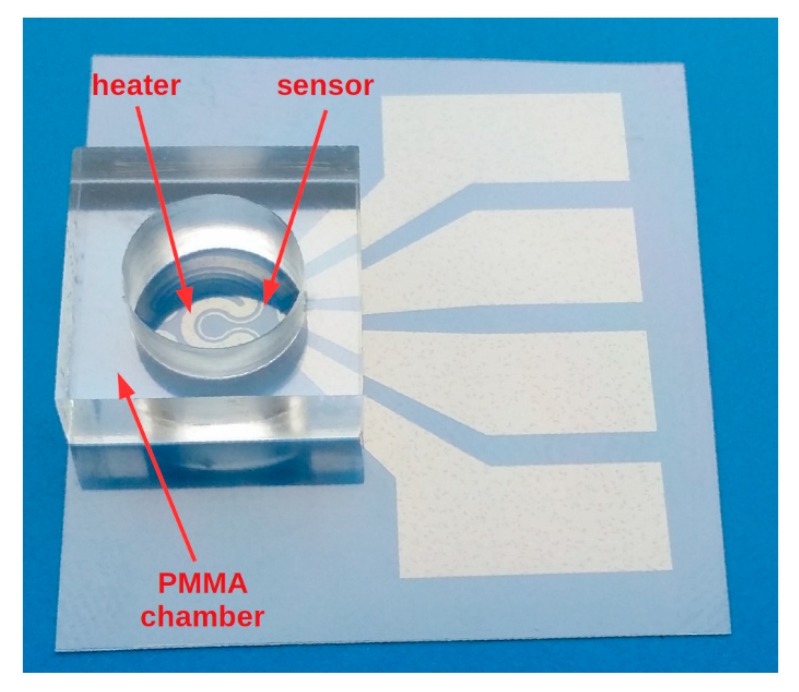
Heater-sensor structure integrated with chamber.

Experimental research was performed to determine the possibilities for practical applications of the presented microstructures. The experimental conditions were designed to be similar to the configuration of eventual applications. Perhaps the most obvious application is to use the heaters for controlled and accurate heating of small volumes of liquids (e.g., a single drop). The experiment required structures with a poly (methyl methacrylate) (PMMA) microchamber attached to the surface (Figure 7). In addition, the system was equipped with two miniature thermocouples: the first mounted on the bottom of the chamber (similarly to that integrated with the microstructure) and the second submerged in liquid and with no contact with the chamber walls. This configuration allowed the dynamics of the thermal processes in the microsystem, caused by different heat loads, to be observed. A single drop of 150 µL pure water and the same volume of natural organic oil were used as heat loads. The results obtained for the empty chamber confirmed the slight influence of PMMA chamber mass on thermal processes. The microchamber did not have direct contact with the heater path and was not an additional heat load.

**Figure 8:**
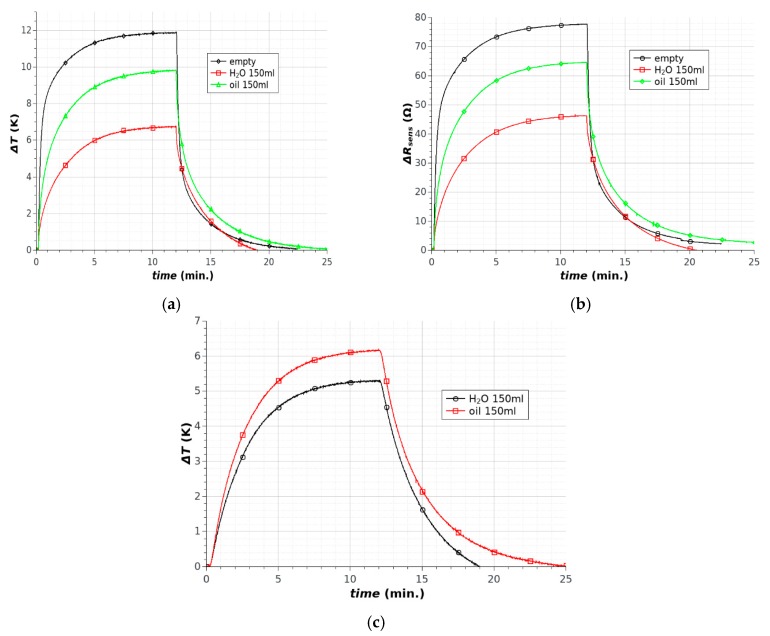
Heating microstructures with chambers at room temperature: (**a**) temperature changes; (**b**) sensor resistance changes; (**c**) temperature changes for liquid inside chambers.

Filling the chamber with liquid allowed the changes in the dynamics of the thermal processes to be observed. The type of liquid played the key role in the kinetics of the thermal processes. The heat capacity and thermal conductivity of water are much higher than those of oil, which significantly changes the dynamics of the observed processes (Figure 8). It should be noted that, as with the results obtained for the microsystem without a chamber, the character of the changes in temperature and sensor resistance were the same. Similar changes in the temperature of the fluids were observed by direct measurement (Figure 8c). The differences in temperature were the result of the position of the sensors. The similar character of the changes is more important. Based on the results, it is possible to use the microsystems for the accurate temperature control of small volumes of various fluids near room temperature. It should be noted that the use of our microheater system did not require additional temperature monitoring because the sensor integrated with the heater structure ensured accurate measurement.

**Figure 9:**
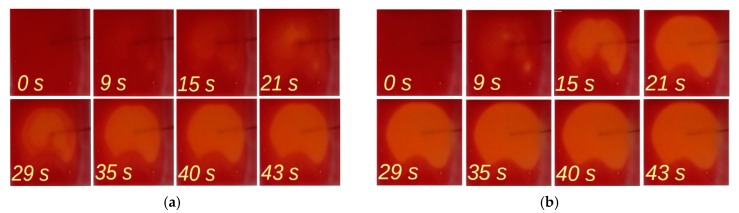
Visualization of the heating of microstructures using a thermochromic coating: (**a**) 100 mA; (**b**) 140 mA.

To visualize the heating process in the microheater due to current flow, images of the structure covered with a thin layer of thermochromic varnish were recorded. Figure 9 provides a comparison of images of the heating structure with different currents for the same length of time. As expected, the heating process was much shorter with higher current (140 mA) than with a current of 100 mA. After some tens of seconds, the temperature distribution of the heater structure became homogenous. The small horizontal shadow visible in the right side of the images was produced by the thermocouple attached to the reverse side of the structure.

### 3.2. Cryogenic Temperature

**Figure 10:**
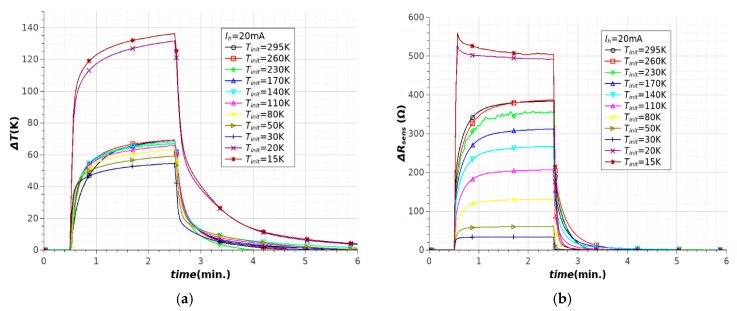
Heating of microstructures at cryogenic temperatures: (**a**) temperature changes; (**b**) sensor resistance changes.

**Figure 11:**
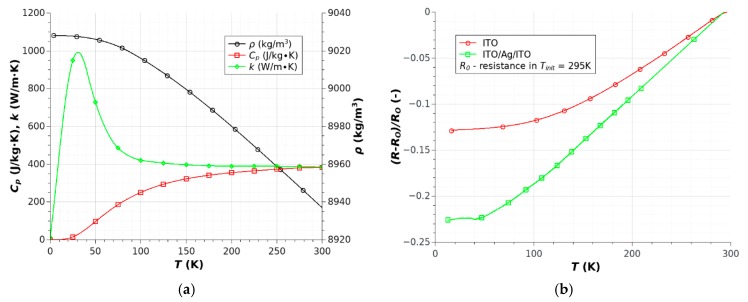
Temperature dependence of material properties: (**a**) copper; (**b**) TCO layers.

Time dependences of temperature changes ΔT and sensor resistance changes ΔR were observed at cryogenic temperatures (Figure 10). Experiments were conducted at several different initial temperatures, ranging from 15 K to 295 K. An ITO/Ag/ITO layer was used because the resistance changes of this layer were linear at a wide range of temperatures, from 300 K to 50 K (Figure 11b). The resistance changes of the ITO layer were smaller, especially below 150 K, which prevented use of a coupled temperature sensor. The additional heat load (copper heat exchanger) and lower temperature allowed the use of higher heat power in these studies (1 W for I_h_ = 20 mA). In addition, the contact cooling method efficiently eliminated local hotspots in the heater path, which allowed higher heat power to be used. In contrast to those obtained at room temperature, the results at cryogenic temperatures were more varied and more difficult to interpret. The wide range of temperatures changed all the properties of the heat exchanger and microsystem significantly. The most important changes were observed at temperatures below 25 K (Figure 10). For the same heat power (about 1 W), different temperature changes were measured in a range below 25 K and higher than 25 K. It should be noted that this was not a result of rapid changes in the TCO electrical properties (Figure 11a). For temperatures below 150 K to 15 K, the ITO resistance changes were very small at <3%. The resistance changes in the ITO/Ag/ITO layer were linear in the range 300–50 K and nearly constant below 50 K. For this reason, the sensor resistivity changes were not linearly dependent on the temperature changes, especially below 50 K for ITO/Ag/ITO. Noticeable changes of copper thermal properties were observed below 100 K to 30 K. In this range of temperature, the heat transfer from structure to heat exchanger increased slightly which caused decreasing structure temperature (Figure 10a, curves for T_init_ = 80 K, 50 K and 30 K). The step changes in the thermal properties of the copper heat exchanger were responsible for an unconventional increase in temperature of structures below 30 K. The rapid decreases in heat capacity (c_p_) and heat conductivity (k) were noted in this range (Figure 11a). It should be noted that copper heat capacity was reduced to a very low value, close to zero. The strong decrease of heat capacity meant a rapid reduction of heat load. This limited the heat transfer from heater to the heat exchanger and consequently to the second stage of the cryocooler. For the same current flowing in the heater (Figure 10a) the temperature of structure reached a higher value (Figure 10a, curves for T_init_ = 20 K and 15 K). The heat capacity of copper exchanger determined the thermal phenomena in temperatures below 30 K.

The results confirm the possibility of controlling the temperature of small-scale cryogenic systems using the TCO heating microsystem.

## 4. Conclusions

This paper has presented an analysis of the electrical and thermal properties of transparent heaters integrated with temperature sensors, formed in transparent oxides layers deposited on polymer substrates. Both the heating structures and the sensors were produced by laser ablation in one technological process and could have any repeatable geometry. The minimal transverse dimensions of the sensor path were about 100 µm, equal to the distance between the sensors and the heater path, so each was under the same thermal conditions. Therefore, no other form of temperature measurement is necessary. The surface power of the heater reached about 3 W/cm^2^. The microheater exhibited superb heating and cooling dynamics with and without a heat load. The special features of the proposed microheater include its ability to operate at cryogenic temperatures, especially when produced in an ITO/Ag/ITO layer. The resistance changes of the ITO/Ag/ITO layer were linear in the range 300–50 K and nearly constant below 50 K. These advantages provided easier measurement and control of temperature from 50 K to 350 K. When integrated with polymer chambers, the microheaters could be applied for controlled and accurate heating of small volumes of any liquid. The microheater could be applied in many research and control processes, especially when it is problematic to test larger volumes of a medium. It is possible to manufacture a series of microheaters which could be heated and controlled independently. The high repeatability and precision of the heater-sensor structures and low cost of preparation allow for single use applications of the devices.

## Figures and Tables

**Table 1:** Heaters based on transparent oxides.

No.	Heater Material; Thickness	Substrate	Shape of Heater; Preparation Method	Surface Area (mm^2^)	Surface Power (W/cm^2^)	Refs.
1	ITO; 100 nm	glass	rectangle; vacuum deposition	72	— *	[16]
2	ITO; —	glass	square; photolithography and etching	0.01	≈133	[17]
3	ITO; 300 nm	glass	square; vacuum deposition and etching	255 ÷ 413	5 ÷ 11	[18]
4	ITO; 500 nm	quartz glass	rectangle; lift-off technique deposition	≈150	—	[19]
5	ITO/Ag; 100 nm	glass	outer surface of tube; sputtering	125 ÷ 565	≈4	[20]
6	ITO; —	quartz glass tube	outer surface of tube; —	≈628	2.18	[21]
7	ITO; —	glass tube	outer surface of tube; vacuum deposition	628 ÷ 12,000	0.2 ÷ 4	[22]
8	ITO; —	glass	rectangle; patterned by photolithography	≈6500	—	[23]
9	ITO nanoparticle solution; 470 nm	glass	rectangle; spin coating and sintering	≈350	≈0.6	[2]
10	ITO; 350 nm	sapphire	rectangle; sputtering	640	50–134	[24]
11	ITO; —	sapphire tube	outer surface of tube; sputtering	15,000	—	[25]
12	ITO; 180 nm	pyrex glass	square; —	25	5.89	[26]
13	ITO; —	pyrex glass	square; etching	1	300	[27]
14	ITO; 140 nm	Si_3_N_4_	square; photolithography and etching	~0.67	17.8	[28]
15	Ga-doped ZnO; 400 nm	glass	rectangle; photolithography and etching	110	≈1.32	[29]
16	ITO/Ag/ITO; 40/6 ÷ 12/40 nm	PET	rectangle; cutting of large area film	2500	0.28 ÷ 0.33	[30]
17	ITO; 170 nm	glass	square; photolithography and etching	4	1.9 ÷ 6.25	[31,32]
18	ITO; 170 nm	glass	square; photolithography and etching	5.52	4.55 ÷ 10	[33]
19	ITO; 170 nm	glass	square/meander; photolithography and etching	5.52	10	[34]

* — no data.
